# Supplementary material for: Patterns of Intron Gain and Loss in Fungi
Source: PLoS Biol. 2004 Nov 30;2(12):e422. doi: 10.1371/journal.pbio.0020422 (PMC532390; doi:10.1371/journal.pbio.0020422)
Supplement: Table S1 — Also available at http://genes.mit.edu/NielsenEtAl/. (4.3 MB ZIP). [file pbio.0020422.st001.zip › NielsenEtAl/html/981.html]

AN6004.1.NCU05488.1.MG00444.1.FG08365.1


```
 CLUSTAL W (1.82) Multiple Sequence Alignments - Introns Inserted


Sequence 1: NCU05488.1	283 aa
Sequence 2: MG00444.1	281 aa
Sequence 3: FG08365.1	277 aa
Sequence 4: AN6004.1	270 aa
Alignment Length: 296 aa
Number Identitical Residues: 92 aa
Alignment Score (without introns) 4916


MG00444.1 	MSN--TVTVKNISAKTDDKEIRDFFAFC2GKITDLQVN-TEGE---TKSATVIFEKET~A
NCU05488.1	MS---TVYVKNIGANTEEKDIRAFFSFC2GKISSLDVT-TEGE---TKSATVTFEKES~A
FG08365.1 	MSANSTINVKNIAPATSDSEIKDFFSFC2GKIQDLKVS-QDGE---TKSAEVIFEKET~A
AN6004.1  	MSDRTTVHVSNIDPSTSEKEVQDFFSFC2GKIVSISVTPTSGEPGSLKSATVTFEKDA2A
          	**  :*: *.** . *.:.::: **:** *** .:.*.. .**..: *** * ***:: *

MG00444.1 	AKTALLLNNTQLGPSHISVTSDS----PVGDGAHPEKTNADRDSDEITQEDKPRARILAE
NCU05488.1	ARTALLLDHTKLGEHELSVTSASGEHADSGDNVHP-KSDADRDTDEITQEEKPRARVLAE
FG08365.1 	KKTALLLNNTQLGTNHLEVTSAT----ADGEDDVPTTKNTDRDSDEITQEEKPRARILAE
AN6004.1  	AKTALLLDQTQLGPSVVTVKAAQTLDEIAGEHSATAAEAKDENQNDLEQEDKPRSRIIAE
          	 :*****::*:**   : *.:        *:   .     *.: ::: **:***:*::**

MG00444.1 	YLAHGYVVGDAAIQKAIDLDQHHGVSSRFVGTLQNLDQKYHATDRAKTADQSYGITTRAT
NCU05488.1	YLASGYLVADSGLKTAIALDEKHGVSQRFLSTIQNLDQKYHATDRAKTADQSYGITARAN
FG08365.1 	YLAHGYVVGDAAIQRALDLDATHGVTNRFFSTLKGLDEKYHATDRAKATDESYGISQRAN
AN6004.1  	YLAHGYTISDQAIQKAIAIDKKHGFTSRFTSVLSNFDKKTGASERAKGLDESYKISDKAA
          	*** ** :.* .:: *: :*  **.:.** ..:..:*:*  *::***  *:** *: :* 

MG00444.1 	SLLTGINSYFEKAASTPTGKKIVDFYTQSSKQVQDIHAEAVRLAELKKQDAGGSAARAAG
NCU05488.1	SLFSGLSSYFEKALEAPGAKKIVDFYTTGSKQVQDIHNEAKRLAELKKQEAGGSSYKAAG
FG08365.1 	SMWLGLSSYFEKASNTPTGKKIAEFYTEGSRQVQEVHAEARRLADLKKEEHGGSAYKAAG
AN6004.1  	NSWRGLHSYFEKAINTPSGRKLRDFYSQTDKQVRDIHAEARRLADLK---AGKTEEKKAE
          	.   *: ****** .:* .:*: :**:  .:**:::* ** ***:**    * :  : * 

MG00444.1 	LEKILGTSAAGTEKPKDGASDSPAAVPGTVPASGGAT-TTEKPKTNPVEP--DPKV--
NCU05488.1	LDKIFG-----AEKAPGQESKPNDQVPGAAPSDAAATESNQQPISEGAYPGTAEKIPQ
FG08365.1 	LEKVFG-----KEKTSG--TAAPEAGHGALGTSVPAVSADNGPNVSAPEAG--EKH--
AN6004.1  	GE---------SSSPATESNPAPAPAPGVEPEAAQAAPATTEKA--------------
          	 :          ...    . .     *.      *. :
```
